# Looking through *Staphylococcus pseudintermedius* infections: Could *Sp*A be considered a possible vaccine target?

**DOI:** 10.1080/21505594.2018.1426964

**Published:** 2018-03-19

**Authors:** Erika Grandolfo

**Affiliations:** Department of Veterinary Medicine, University of Bari “Aldo Moro”, Valenzano, Italy

**Keywords:** *Staphylococcus pseudintermedius*, *Sp*A protein, vaccine development

*Staphylococcus pseudintermedius* is the most prevalent inhabitant of the skin and mucosa of dogs and cats, and also the major bacterial pathogen causing canine skin and ear infections [1–2]. It was firstly described in 2005 based on 16S rRNA gene sequencing analysis of isolates from a cat, a dog, a horse, and a parrot [3], then included in the SIG group within *S. intermedius* and *S. delphini* [4]. *S. pseudintermedius* can be isolated from the nares, mouth, anus, groin and forehead of healthy dogs and cats, as well as from dogs and cats with inflammatory skin disease [1,5]. In recent years, there has been an increasing number of infections reported worldwide in dogs and cats caused by methicillin-resistant *S. pseudintermedius* (MRSP) [6,7], especially associated with two predominant clones, sequence types (STs) 71 and 68 [8–11]. Infections caused by these two MRSP clones are difficult to manage due to their multidrug-resistant (MDR) profiles [8,12]. Furthermore, the MDR ST71-MRSP lineage was reported in a human infection for the first time in Switzerland in 2010 [13] and subsequently reported in owners of infected pets [14], showing that, although it is estimated to be low, there is risk of zoonotic transmission.

The successful fitness of *S. pseudintermedius* is due to several potential virulence factors, such as cell wall-anchored proteins (CWA), exoenzymes and exotoxins [15–16]; these virulence factors have been better studied, and characterized, in *S. aureus* than in *S. pseudintermedius*, although it has been shown that most of them have similar features [17]. This is the case of the *Staphylococcal Protein* A (*Sp*A), a 40–60 kDa protein belonging to the most prevalent group of CWA proteins, i.e. microbial surface component recognizing adhesive matrix molecules (MSCRAMM). In *S. aureus, Sp*A comprises five repeated domains (E, D, A, B, and C), each of them binding with high affinity the Fc region of immunoglobulin (Ig) G and the Fab region of Ig of the variable heavy VH3 subclass. Protein A is expressed by virtually all clinical *S. aureus* isolates [18] and it is secreted during the exponential growth phase [19]. Protein A is immunosuppressive due to its binding activities on human and animal Igs. Protein A binding to the Fc domain of IgG blocks opsonophagocytic killing (OPK) [20], whereas *Sp*A binding to Fab and cross-linking of IgM promotes B cell superantigen activity [21]. Notably, *Sp*A cross-linking of B cell receptors triggers proliferation and apoptotic collapse of the expanded lymphocyte populations [22]. Along with a centenary work on staphylococcal vaccines [23], recently it has been shown that immunization with a nontoxigenic protein A (designated *Sp*A_KKAA_) enabled infected Guinea pigs to elicit a protective antibody response against *S.aureus* infection [24].

In light of these considerations, the study conducted by Balachandran et al [25]. has investigated the common features between *S. aureus* and *S. pseudintermedius* protein A in order to understand if *S. pseudintermedius* protein A could be a candidate for vaccine development. To date, little is known about *S. pseudintermedius Sp*A and the above authors have filled a gap. In fact, a putative *spa* gene was initially described by Moodley et al. in 2009 [26]; subsequently, whole genome sequencing of canine *S. pseudintermedius* ED99 [27] revealed that, within 18 putative genes that encode CWA proteins, namely *S. pseudintermedius surface* (*sps*) genes (from *sps*A to *sps*R), two genes had an orthologous conformation with *S. aureus spa* encoding protein A, namely *sps*P and *sps*Q [27]. The *sps*P gene encodes a protein that consists of 377 amino acids; it has a predicted N-terminal sequence of 33 amino acids, followed by a repeat region consisting of three IgG-binding domains. The C-terminal region has a predicted X-region which shares 63% sequence similarity to the X-region of *S. aureus*. On the other hand, the *sps*Q gene encodes a protein that consists of 462 amino acids, with a predicted terminal sequence of 33 amino acids, followed by a repeat region consisting of four IgG-binding domains. The C-terminal region has a predicted X-region which includes a 77 amino acid-long repeat sequence (Xr) and a constant region (Xc) with 70% similarity to the X-region of *S. aureus* [26]. Riley et al. recently reported at the genomic level that a full-length *sps*Q gene was always present in clinical isolates of *S. pseudintermedius*, while *sps*P gene was harbored less frequently [28]. In this work Balachandran et al. examined 18 clinical *S. pseudintermedius* isolates associated with different STs and demonstrated that *sps*Q gene was present in all the isolates, as already observed [28], but its expression level varied among STs and protein A secreted in the supernatant. The difference was found to be significant for ST71 compared to ST68, and also for these two clones when compared to other sequence types.

These results represent additional information on the worldwide success of these two lineages that are nowadays a considerable challenge for the treatment of MRSP infections. In general, data about *S. pseudintermedius* virulence are scarce and continuous efforts are necessary to better understand *S. pseudintermedius* pathogenicity. During the last decade, the relationship between dogs and their owners has changed dramatically. As a result, transmission of microorganisms between humans and their dogs is increasing, as observed for the MDR ST71, which has been isolated at high frequency both in animals and humans [13,14]. *S. pseudintermedius* resides on the skin or mucosal surfaces of dogs and might easily be transmitted to pet owners either by direct contact or by sharing the same environment in the household [29,30]. The risk of MRSP infection in humans is unclear, but there are evidences of potential long-term carriage of MRSP in humans due to the particular ability of some MRSP lineages, such as ST71 and ST68, to adapt to the human host [31].

Other aspects of protein A not yet clarified in *S. pseudintermedius* and well speculated in *S. aureus* were addressed in this study. In fact, the authors demonstrated that *S. pseudintermedius* protein A is a CWA protein able to bind canine IgG primarily via its Fc region, it is recognized by the anti-protein A raised against *S. aureus* protein A, can be secreted during log phase, and its block makes *S. pseudintermedius* more susceptible to phagocytosis [25]. *S. pseudintermedius Sp*A is a virulence factor that allows the bacterium to evade the host immune response; the primarily interaction with the Fc fraction of IgG hinders phagocytosis, because bacteria coated with IgG in an inappropriate conformation which becomes not recognizable by the Fc receptors on polymorphonuclear cells [20]. This means that *S. pseudintermedius* isolates expressing protein A are able to escape from opsonization and phagocytosis, leading to persistent infections. These results were also confirmed by the phagocytosis assays performed by the authors.

On the other hands, several progresses have been made on immunological aspects of *Sp*A in *S. aureus*. Infections caused by *S. aureus* do not generate protective immunity in either humans and or animals [32]. Vaccines that elicit antibody responses against bacterial envelope components (CP5/CP8, ClfA, or IsdB) with the intent of promoting OPK may have been hindered because immunoglobulin effect or functions are modified by *Sp*A [33]. To date, studies on the preclinical development of vaccines have been performed with encouraging results. For instance, immunization of mice or rabbits with an engineered *Sp*A_KKAA_, in which 20 amino acid residues essential for its association with Ig Fc and Fab were replaced, elicited antibodies that neutralize *Sp*A [24]. The *Sp*A_KKAA_ – derived polyclonal antibodies promoted OPK of staphylococci, suppressed staphylococcal B cell superantigen activity and promoted humoral immune responses against a wide spectrum of antigens [24]. Moreover, these observations were confirmed by studies with *Sp*A_KKAA_ monoclonal antibodies (MAb). *Sp*A_KKAA_ MAb-mediated neutralization of *Sp*A, promoted OPK of *S. aureus* and the development of antibody responses against many different antigens [34]. It has been observed that the broad spectrum of humoral immune responses can prevent the pathogenesis of staphylococcal infections [32]. Recently, it has been shown that *S. aureus* lacking protein A (Δ*spa*), or expressing the nontoxigenic variant, displayed defects in abscess formation and could not suppress the adaptive immune responses of infected animals [35].

These preliminary results underline the importance of the study of Balachandran et al [25]. which paves the ways for *S. pseudintermedius* infections; moreover it is still necessary to determine if there are toxic effects of *S. pseudintermedius* protein A on B cells. The anti-protein A antibody used in this study was raised against *S. aureus* protein A [25], suggesting that the proteins A of these two species share epitopes. In light of these results, it is clear that the analysis of successful candidates for vaccine development against *S. pseudintermedius* should include antigenic targets rapresentative of a wide variety of strains and STs.
